# Construction and Calibration of the NIST Large-Area-Source X-Ray Counting System

**DOI:** 10.6028/jres.096.045

**Published:** 1991

**Authors:** J. M. R. Hutchinson, M. P. Unterweger, P. A. Hodge

**Affiliations:** National Institute of Standards and Technology, Gaithersburg, MD 20899

**Keywords:** calibration, efficiency curve, large area source, plutonium-238, proportional counting, x ray

## Abstract

This paper describes the construction and calibration of the NIST large area x-ray counting system. ^238^Pu sources 8 in (20.32 cm) by 5 in (12.70 cm) thick, emitting *L* x rays in the range of 12–20 keV are calibrated for total emission rate and also for rate through a centrally located 3 in (7.63 cm) diameter aperture. Alpha particle emission rates are obtained using the known x-ray to alpha particle abundances. The sources will be used to calibrate alpha-particle surface monitors.

## 1. Introduction

Recently the National Institute of Standards and Technology (NIST) has developed a calibrated system for measuring the *L* x-ray-counting rate of large area sources of plutonium. The system was requested by the United States Air Force (USAF) primary calibration laboratory in order to calibrate large area standards for its secondary and tertiary laboratories. The objective was to tie the USAF calibrations to NIST standards and thereby take the first step towards “traceability” of USAF surface monitoring equipment.

The program required the construction of a jig which holds a NaI(Tl) detector at a precise distance from a vertically moveable platform on which the large area source may be positioned. Distances from the source-to-detector can be adjusted and held accurately. The second part of the program was to determine the ^238^Pu *L* x-ray counting efficiencies as a function of source-to-detector distance for both a rectangular source and circular sources of various diameters. A number of published calculations and tables exist of photon efficiencies at various distances from NaI(Tl) detectors for these geometries [1]. Generally, though, the accuracies given in these reports are in the range of 5–10 percent for the dimensions of the present arrangement, especially for close-in geometries for which the source dimensions exceed those of the detector by a wide margin. The aim of the program is to provide efficiency values with accuracies of 5 percent or less.

The calibration procedure was first to check the system calibration with a calibrated point source which can give the efficiencies for all distances from an analytical expression of the solid angle subtended by the “black” detector from the source. Values of the count rates for rectangular and circular large area sources at various distances were fitted to a mathematical expression which reduced to the point source values for zero source size. The calibration of one of the sources was then compared with the x-ray results by alpha-particle counting.

The utility of photon measurements for field monitoring is described by the NARP manual [2] which recommends low-energy gamma instruments for field surveys of plutonium contamination. Alpha measurements should be used “primarily for personnel monitoring and when field use is necessary, on smooth surfaces only, e.g., pavement and building surfaces. ”

## 2. Large Area X-Ray Counting System

**Fig. 1:**
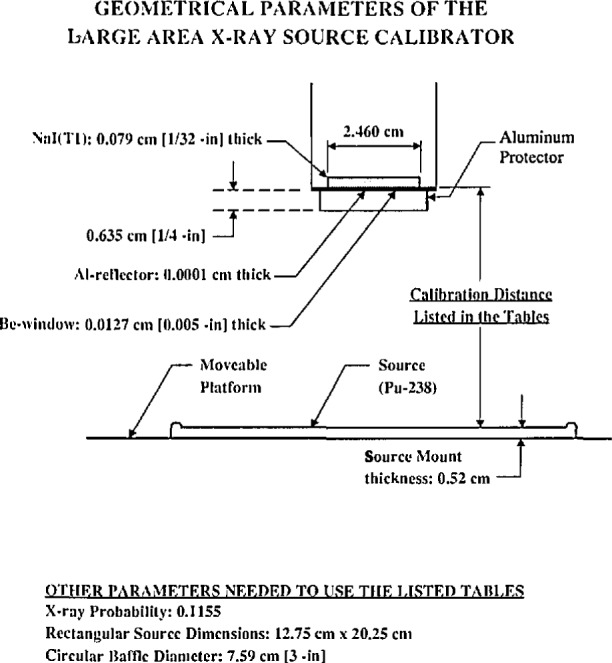
Schematic diagram of the x-ray system.

**Fig. 2:**
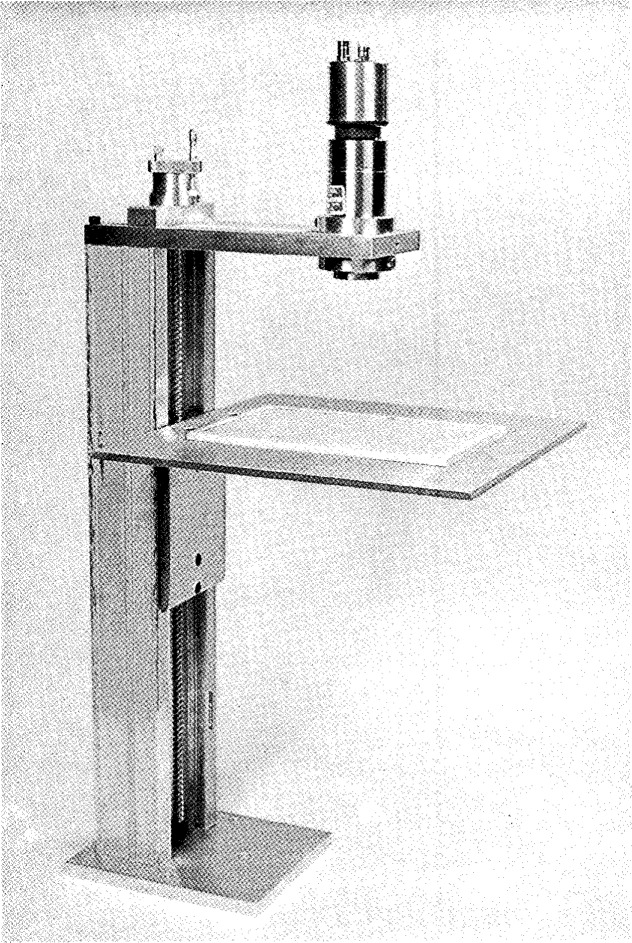
Photograph of the x-ray system.

The large-area-defined-solid angle NaI(Tl) x-ray counting system is shown schematically in Fig. 1 and in a photograph in Fig. 2. It consists of a 1/32 in (0.079 cm)^1^ thick, 31/32 in (2.46 cm) diameter NaI(Tl) detector. The detector is thick enough to ensure that all impinging U *L* x rays (following the alpha decay of ^283^Pu) are absorbed except those which are lost in the 0.005 in (0.013 cm) Be window and the Al reflector. The detector is mounted on an XPIOIO phototube which has a noise level equivalent to one keV photon energy. The platform is vertically moveable over approximately 14 in (35.56 cm) by means of a precision spindle which is moved manually with a wheel which can reproduce the vertical position to 0.001 in (0.003 cm). The source platform is large enough to be able to mount any size source that could be useful for the calibration of surface monitoring systems. Tests were made on the NIST coordinate measuring machine. The true distance of the platform for selected positions is given in Table 1.

The vertical variability from horizontal is approximately 0.014 in (0.036 cm) maximum over the face of the platform. Deviation of the center from the labeled value is 0.002 in (0.006 cm).

**Fig. 3:**
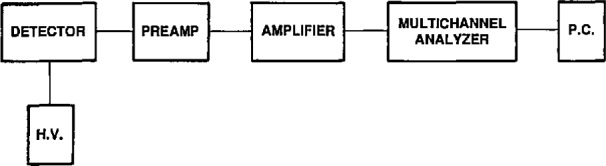
Schematic diagram of the electronics of the x-ray system.

**Fig. 4:**
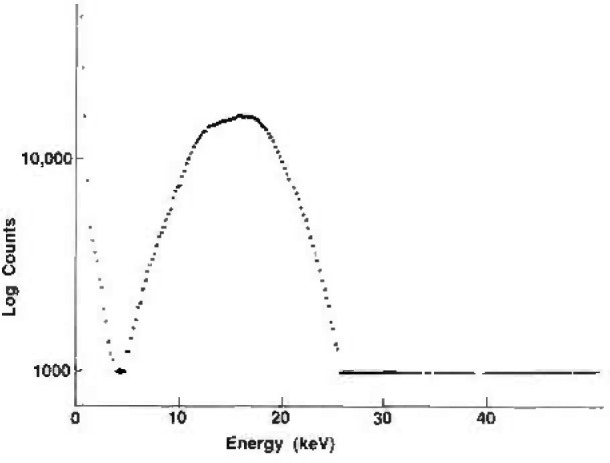
Spectrum of a NaI(Tl) source.

A schematic diagram of the electronics is shown in Fig. 3. The pulses are fed from the amplifier with microsecond resolving time into the pulse height analyzer whose output is recorded on a PC. A typical NaI(Tl) spectrum of the U *L* x rays in the decay of ^238^Pu is shown in Fig. 4.

## 3. Sources and Baffles

**Fig. 5:**
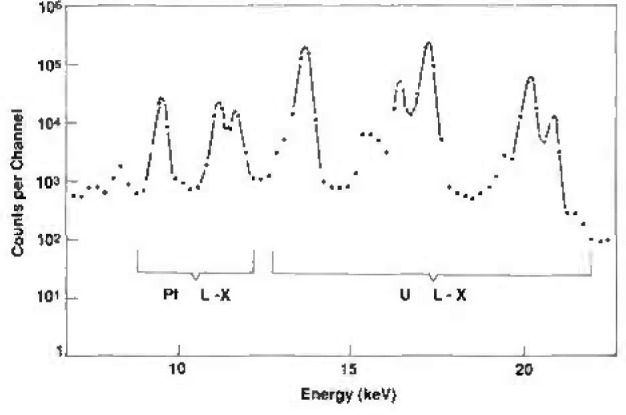
Ge spectrum of a ^241^Am point source electroplated on a platinum substrate.

A NIST “point” source was used to check for the correct functioning of the system and to determine the efficiency of the detector for x rays at large distances. It consists of ^238^Pu electroplated onto a thin platinum foil 6 mm in diameter and mounted onto a 1 in (2.54 cm) diameter polished stainless steel disc. The photon emission spectrum with a Ge detector from this source is shown in Fig. 5. The U *L* x-ray peaks occur between 11 and 20 keV. Contributions from activation of the platinum occur at 8 and 11 keV and are 7±2 percent of the intensity of the U *L* x rays. In addition, the possibility exists for self activation of the source, i.e., high energy *L* x rays stimulating lower energy x rays in the plutonium or being lost entirely from the photon spectrum. Because the solid angle from source to detector can be calculated exactly (within the accuracies of the measured dimensions of the source-to-detector distance and diameter of the detector) and the source activity is known, the correct functioning of the system was checked with measurements with this source.

**Fig. 6:**
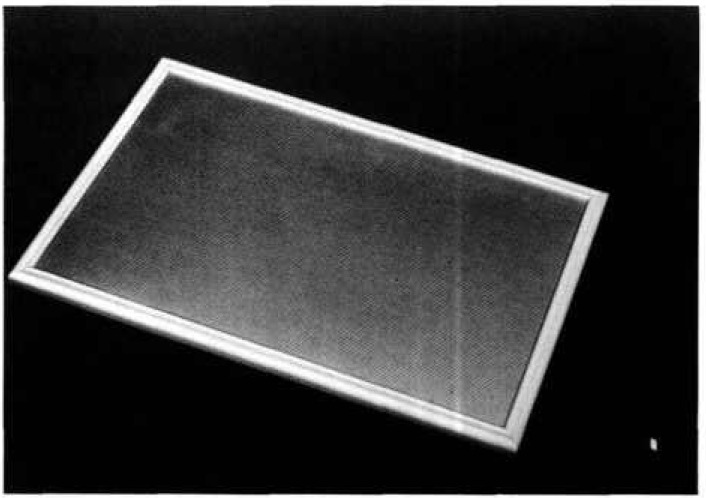
Large area ^238^Pu source.

**Fig. 7:**
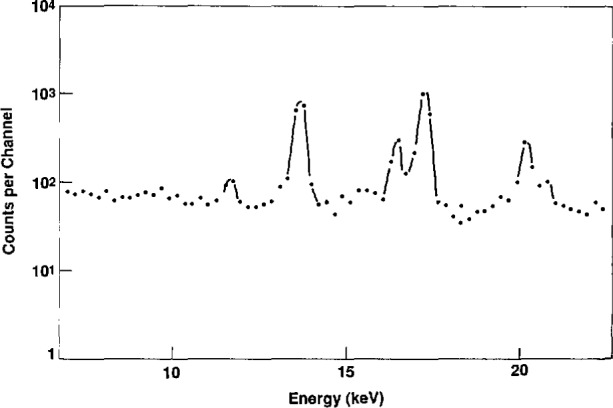
Ge spectrum of the large area ^238^Pu source.

**Fig. 8:**
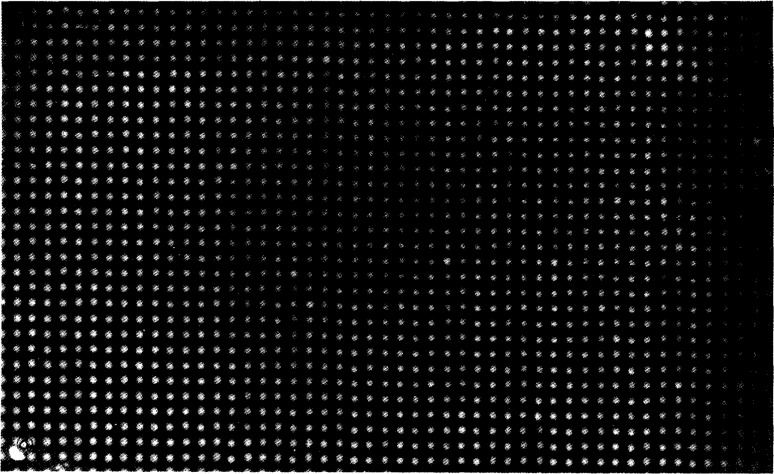
Radiograph of the large area source showing an array of radioactive sites.

A large area source is shown in Fig. 6. The photon emission spectrum with a Ge detector from this source is shown in Fig. 7. The dimensions are 8 in (20.32 cm) by 5 in (12.70 cm). Disc sources were produced by covering it and other similar sources with baffles of 1 in (2.54 cm), 2 in (5.08 cm), and 3 in (7.62 cm) diameter holes cut in either stainless steel or aluminum (6061) plates. The plutonium is anodized onto the aluminum foil which is attached to a solid aluminum baseplate. A radiogram is shown of one of the “hotter” sources showing an array of radioactive sites, 4 mm distance center-to-center (Fig. 8). The weaker sources were also anodized but, in these, the radioactivity is continuously distributed. The observed homogeneity of these sources has been discussed elsewhere [3] and was found to be in the range of ±5 percent for a 3 in (7.62 cm) diameter area.

## 4. X-Ray Efficiency Curve

### 4.1 Point Source

Measurements were taken of the point source with the Nal(Tl) detector at distances from 1 in (2.54 cm) to 7 in (17.78 cm) from the source to the face of the detector.

The expression for the solid angle from a point source at a distance *h* to a circular aperture with radius, *r*, is:

ϵ=0.5−h/2R (1)

where ϵ is the solid angle, or in this case, the efficiency, and *R*^2^ *= r*^2^ *+ h*^2^. *A* correction was made for the scattering by the intervening air, and absorption by the Be window and the Al reflector.

The experimental value appears to be 5 percent greater than the calculated value. However 7±2 percent of the NaI(Tl) peak has been determined to be stimulated x rays from the platinum substrate. Subtracting this value leaves the experimental value in good agreement with the prediction in Eq. (1).

### 4.2 Large Area Sources

The value for the activity of source AA372, the most active of the sources, was determined two ways:

- By taking the count rate at 14 in (35.56 cm) with a 3 in (7.62 cm) disc baffle and assuming that it was essentially a point source at that distance and computing the efficiency according to Eq. (1) (adjusting for air and Be and Al reflector absorption). The result was multiplied by the ratio of the total rectangular area to the area of the 3 in disc to obtain the total activity, and
- By alpha-particle counting AA370 [3], a relatively low activity source that would not overload the internal gas counting system, and comparing AA370 and AA372 by x-ray counting and external alpha counting, and from these data determining the activity of AA372. A source self-absorption correction of 5.8 percent was determined as follows. (It should be remembered that the source is anodized so that the active material is embedded in aluminum oxide and significant absorption is to be expected.)

**Fig. 9:**
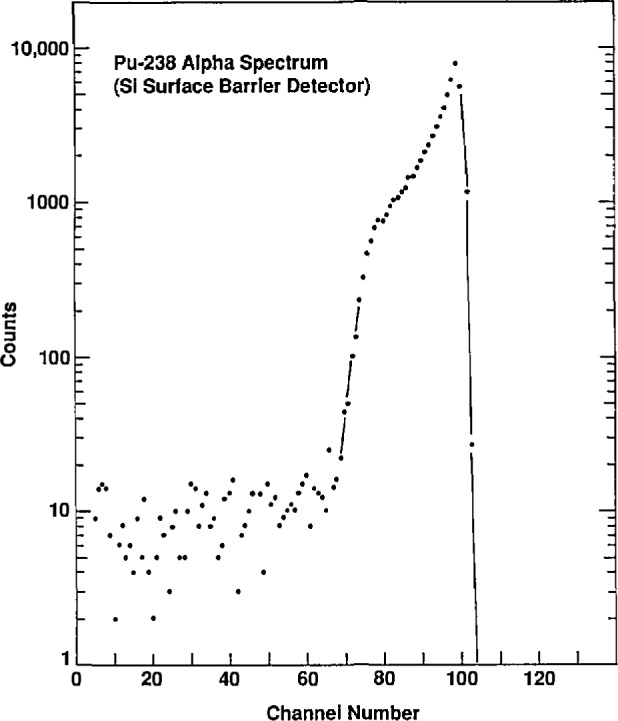
Si surface barrier spectrum of a large area ^238^Pu source.

A Si surface barrier spectrum as shown in Fig. 9 was taken. The distribution, except for a small amount of tailing, starts at 80 percent of the range. This is because the activity at its deepest is impregnated to a depth corresponding to 20 percent of the range. The *C*_2*π*_/*N*_0_ value for this situation has been calculated by Lucas et al. [4]. However, the spectrum peaks at 100 percent of the range and tails off at lower energies corresponding to a depth profile for the activity of maximum near the surface and tailing off to a maximum depth of 20 percent of the range.

To determine the overall *C*_2π_*/N*_0_ value, values of *C*_2π_*/N*_0_ at 80, 85, 90, 95, and 100 percent of the range were calculated. Counts in a rectangular area corresponding to 80–100 percent of the range were calculated from the pulse height spectrum and assigned a corresponding *C*_2π_*/N*_0_ value. This fraction of the spectrum was subtracted, and the 85 percent fraction was then calculated, etc. The overall *C*_2π_*/N*_0_ was taken as the weighted average. The weighted sets of *C*_2π_*/N*_0_ were added together with the result that the average value of *C*_2π_*/N*_0_ for the large area source was 0.473, or a correction to the activity of 5.8 percent which agreed with the manufacturer’s value of 5.5 percent. The overall results are shown below.

Activity of AA372 ^238^Pu Source by Two Methods

#### X-Ray Counting

$$\begin{array}{l} DPS=N\;f\;(1/\epsilon )\;(R/0.1155)\\ \;=(4.3141\;(1/0.299\times 10^{-3})\;(5.70/0.1155)\\ \;=7.12\times 10^{5}\;(5/4/91) \end{array}$$

where

*N* = cps at 14 in (35.56 cm)
*f* = x-ray absorption in air, Be, Al
*ϵ* = geometrical point source efficiency at 14 in (35.56 cm) (*L*x/dps) = 0.1155

#### Alpha Particle Counting

$$\begin{array}{l} DPS=C\times 2\times S\times (372/370)\times D\\ \;=2459\times 2\times 1.058\times 148\times 0.963\\ \;=7.41\times 10^{5} \end{array}$$

where

*C* = 2πα count rate of 370 (7/31/86) 2 to convert to 4π
*S* = scattering correction
(372/370) = measured ratio by x-ray counting
*D*=decay to 5/4/91

The alpha results differ from the x-ray results by 4 percent, a value not outside the range of possible uncertainties as discussed in the next section.

U x-ray efficiency curves were then generated for the rectangular and disc shaped sources by first taking data at 1 in (2.54 cm) through 14 in (35.56 cm) for these geometries. The data corrected for air, Be, and Al absorption were fitted using a χ^2^ minimization technique which generated the following equations for the efficiency for the 3 in (7.62 cm) disc and the rectangular sources.

Circular source:

$$\epsilon_{\mathrm{cir}}=\epsilon_{\mathrm{pt}}+(0.5-\epsilon_{\mathrm{pt}})(-0.23095)\cdot {\left(\frac{R}{r}\right)}^{2}\cdot \frac{r^{4}}{{\left(h^{2}+r^{2}\right)}^{2}}+(0.5-\epsilon_{\mathrm{pt}})(0.04236)\cdot {\left(\frac{R}{r}\right)}^{4}\cdot \frac{h^{2}\cdot r^{6}}{{\left(h^{2}+r^{2}\right)}^{4}}$$ (2)

where *R* = radius of source in cm (3.795 cm).

Rectangular source:

$$\epsilon_{\mathrm{rec}}=\epsilon_{\mathrm{pt}}+(0.5-\epsilon_{\mathrm{pt}})(-0.17197)\cdot \frac{\left(a^{2}+b^{2}\right)}{{\left(2r\right)}^{2}}\cdot \frac{r^{4}}{{\left(h^{2}+r^{2}\right)}^{2}}+(0.5-\epsilon_{\mathrm{pt}})(0.03370)\cdot \frac{\left(a^{4}+(10/9)\cdot (a^{2}\cdot b^{2})+b^{4}\right)}{{\left(2r\right)}^{4}}\cdot \frac{h^{2}\cdot r^{6}}{{\left(h^{2}+r^{2}\right)}^{4}}$$ (3)

where *a* and *b* are the length and width of the rectangular source in cm (*a* = 20.3 cm, *b* = 12.725 cm) and where

$$\epsilon_{\mathrm{pt}}=\frac{\left(0.5-h\right)}{\left(2\cdot {\left(h^{2}+r^{2}\right)}^{1/2}\cdot 1.07\right)}$$ (4)

where

*h* = distance from detector to source in cm
*r* = radius of detector in cm (1.230312).

The total efficiency curves,

$${\left(\epsilon_{\mathrm{cir}}\right)}_{\text{total}}=\epsilon_{\mathrm{cir}}\;f$$

and

$${\left(\epsilon_{\mathrm{rec}}\right)}_{\text{total}}=\epsilon_{\mathrm{rec}}\;f$$

where *f*=x-ray absorption in air, Be, and Al are shown in Figs. 10a and 10b and the total efficiency values for the measured distances are listed numerically in Tables 2 and 3.

## 5. Uncertainty Listing

The uncertainties in the value for the efficiencies in curves are as follows:

**Table:** 

	Percent
Activity of point source	1.0
Estimate of percentage of Pt x rays	2.0
X-ray abundance	1.5
Air, Be, Al absorption estimates	1.5
Uncertainties in fitted parameters	1.5
Counting uncertainties	0.2
Source positioning	0.1
Total 1 s.d. (uncertainties taken in quadrature)	3.4

## Figures and Tables

**Fig. 10a:**
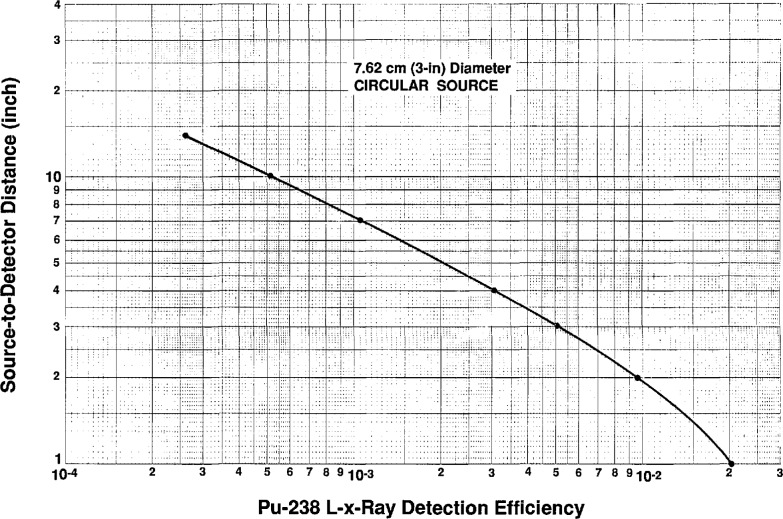
X-ray efficiency curve for the 3 in (7.62 cm) disc source as a function of distance from the front face of the NaI(Tl) detector.

**Fig 10b:**
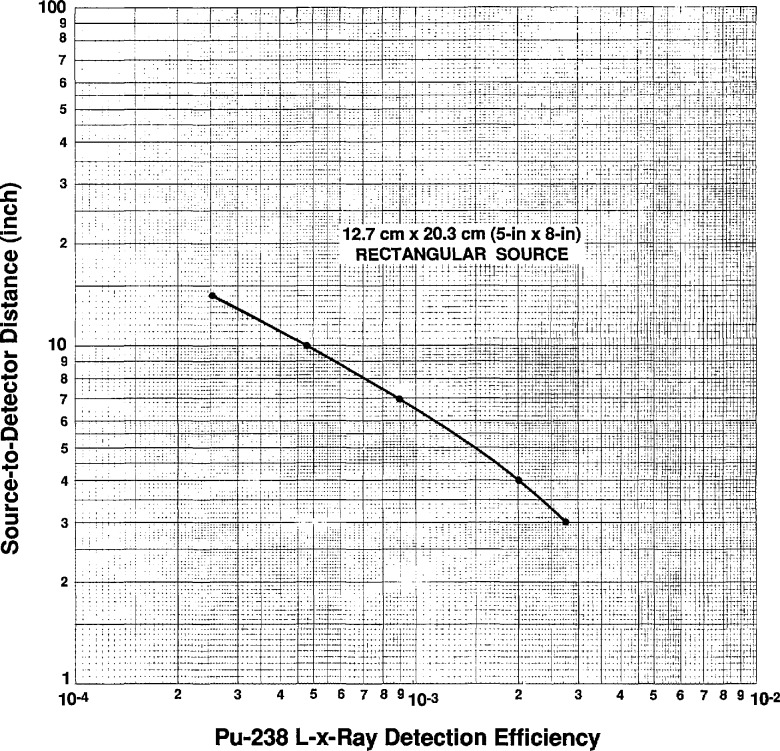
X-ray efficiency curve for the 8 in (20.32 cm) × 5 in (12.70 cm) rectangular source as a function of distance from the front face of the NaI(Tl) detector.

**Table 1:** Coordinate measurement data showing setting accuracies of the platform movement with the spindle

Dial wheel setting		Distance measurement from 0.000 setting
(in)	(cm)	
.000	(0.0)	0.000
1.000	(2.54)	0.9990
2.000	(5.08)	2.0000
3.000	(7.62)	2.9990
4.000	(10.16)	4.0000
5.000	(12.70)	4.9995
6.000	(15.24)	6.0010
7.000	(17.78)	6.9995
8.000	(20.32)	8.0005
9.000	(22.86)	9.0010
10.000	(25.40)	10.0010
11.000	(27.94)	11.0020
12.000	(30.48)	12.0020
13.000	(33.02)	13.0010
14.000	(35.56)	14.0000

**Table 2:** Numerical listing of the calculated and measured efficiencies for the 3 in diameter baffled source

Source to detector distance in in	Calculated efficiency	Measured efficiency
1.00 ( 2.54 cm)	2.359×10^−2^	2.360×10^−2^
1.25 ( 3.18 cm)	1.951×10^−2^	
1.50 ( 3.81 cm)	1.602×10^−2^	
1.75 ( 4.45 cm)	1.317×10^−2^	
2.00 ( 5.08 cm)	1.089×10^−2^	1.081×10^−2^
2.25 ( 5.72 cm)	9.086×10^−3^	
2.50 ( 6.35 cm)	7.660×10^−3^	
2.75 ( 6.99 cm)	6.522×10^−3^	
3.00 ( 7.62 cm)	5.607×10^−3^	5.634×10^−3^
3.25 ( 8.26 cm)	4.863×10^−3^	
3.50 ( 8.89 cm)	4.252×10^−3^	
3.75 ( 9.53 cm)	3.746×10^−3^	
4.00 ( 10.16 cm)	3.322×10^−3^	3.360×10^−3^
4.25 ( 10.80 cm)	2.965×10^−3^	
4.50 ( 11.43 cm)	2.661×10^−3^	
4.75 ( 12.07 cm)	2.400×10^−3^	
5.00 ( 12.70 cm)	2.175×10^−3^	
5.25 ( 13.34 cm)	1.979×10^−3^	
5.50 ( 13.97 cm)	1.809×10^−3^	
5.75 ( 14.61 cm)	1.659×10^−3^	
6.00 ( 15.24 cm)	1.526×10^−3^	
6.25 ( 15.88 cm)	1.409×10^−3^	
6.50 ( 16.51 cm)	1.304×10^−3^	
6.75 ( 17.15 cm)	1.211×10^−3^	
7.00 ( 17.78 cm)	1.127×10^−3^	1.141×10^−3^
7.25 ( 18.42 cm)	1.051×10^−3^	
7.50 ( 19.05 cm)	9.829×10^−4^	
7.75 ( 19.69 cm)	9.208×10^−4^	
8.00 ( 20.32 cm)	8.644×10^−4^	
8.25 ( 20.96 cm)	8.129×10^−4^	
8.50 ( 21.59 cm)	7.658×10^−4^	
8.75 ( 22.23 cm)	7.227×10^−4^	
9.00 ( 22.86 cm)	6.830×10^−4^	
9.25 ( 23.50 cm)	6.465×10^−4^	
9.50 ( 24.13 cm)	6.127×10^−4^	
9.75 ( 24.77 cm)	5.816×10^−4^	
10.00 ( 25.40 cm)	5.527×10^−4^	5.563×10^−4^
10.25 ( 26.04 cm)	5.258×10^−4^	
10.50 ( 26.67 cm)	5.009×10^−4^	
10.75 ( 27.31 cm)	4.776×10^−4^	
11.00 ( 27.94 cm)	4.559×10^−4^	
11.25 ( 28.58 cm)	4.357×10^−4^	
11.50 ( 29.21 cm)	4.167×10^−4^	
11.75 ( 29.85 cm)	3.989×10^−4^	
12.00 ( 30.48 cm)	3.822×10^−4^	
12.25 ( 31.12 cm)	3.666×10^−4^	
12.50 ( 31.75 cm)	3.518×10^−4^	
12.75 ( 32.39 cm)	3.379×10^−4^	
13.00 ( 33.02 cm)	3.248×10^−4^	
13.25 ( 33.66 cm)	3.125×10^−4^	
13.50 ( 34.29 cm)	3.008×10^−4^	
13.75 ( 34.93 cm)	2.897×10^−4^	
14.00 ( 35.56 cm)	2.793×10^−4^	2.810×10^−4^

**Table 3:** Numerical listing of the calculated and measured efficiencies for the 8×5 in source

Source to detector distance in in	Calculated efficiency	Measured efficiency
3.00 ( 7.62 cm)	3.094×10^−3^	3.904×10^−3^
3.25 ( 8.26 cm)	2.827×10^−3^	
3.50 ( 8.89 cm)	2.611×10^−3^	
3.75 ( 9.53 cm)	2.421×10^−3^	
4.00 ( 10.16 cm)	2.247×10^−3^	2.250×10^−3^
4.25 ( 10.80 cm)	2.088×10^−3^	
4.50 ( 11.43 cm)	1.940×10^−3^	
4.75 ( 12.07 cm)	1.804×10^−3^	
5.00 ( 12.70 cm)	1.679×10^−3^	
5.25 ( 13.34 cm)	1.564×10^−3^	
5.50 ( 13.97 cm)	1.458×10^−3^	
5.75 ( 14.61 cm)	1.361×10^−3^	
6.00 ( 15.24 cm)	1.273×10^−3^	
6.25 ( 15.88 cm)	1.191×10^−3^	
6.50 ( 16.51 cm)	1.117×10^−3^	
6.75 ( 17.15 cm)	1.048×10^−3^	
7.00 ( 17.78 cm)	9.854×10^−4^	9.800×10^−4^
7.25 ( 18.42 cm)	9.276×10^−4^	
7.50 ( 19.05 cm)	8.743×10^−4^	
7.75 ( 19.69 cm)	8.252×10^−4^	
8.00 ( 20.32 cm)	7.798×10^−4^	
8.25 ( 20.96 cm)	7.379×10^−4^	
8.50 ( 21.59 cm)	6.990×10^−4^	
8.75 ( 22.23 cm)	6.630×10^−4^	
9.00 ( 22.86 cm)	6.296×10^−4^	
9.25 ( 23.50 cm)	5.985×10^−4^	
9.50 ( 24.13 cm)	5.695×10^−4^	
9.75 ( 24.77 cm)	5.425×10^−4^	
10.00 ( 25.40 cm)	5.173×10^−4^	5.188×10^−4^
10.25 ( 26.04 cm)	4.938×10^−4^	
10.50 ( 26.67 cm)	4.717×10^−4^	
10.75 ( 27.31 cm)	4.511×10^−4^	
11.00 ( 27.94 cm)	4.317×10^−4^	
11.25 ( 28.58 cm)	4.135×10^−4^	
11.50 ( 29.21 cm)	3.964×10^−4^	
11.75 ( 29.85 cm)	3.803×10^−4^	
12.00 ( 30.48 cm)	3.651×10^−4^	
12 75 ( 31.12 cm)	3.508×10^−4^	
12.50 ( 31.75 cm)	3372×10^−4^	
12.75 ( 32.39 cm)	3.245×10^−4^	
13.00 ( 33.02 cm)	3.124×10^−4^	
13.25 ( 33.66 era)	3.009×10^−4^	
13.50 ( 34.29 cm)	2.901×10^−4^	
13.75 ( 34.93 cm)	2.798×10^−4^	
14.00 ( 35.56 cm)	2.700×10^−4^	2.733×10^−4^

## Footnotes

1 The dimensions are given in U.S. customary units with their SI equivalents in parentheses because the instrument is designed and calibrated in inches and fractions thereof.
